# Impact of iN2L Tablets on Loneliness and Well-Being: Findings of an Innovative Industry-AAA Program

**DOI:** 10.1093/geroni/igab046.3399

**Published:** 2021-12-17

**Authors:** Lydia Nguyen, Karen O'Hern, Adam Siak, Kristi Stoglin, Charlotte Mather-Tayor

**Affiliations:** 1 iN2L, Denver, Colorado, United States; 2 iN2L, Greenwood Village, Colorado, United States; 3 Area Agency on Aging of Broward County, Sunrise, Florida, United States

## Abstract

Area Agency on Aging (AAA) senior and adult day centers closed due to COVID-19, causing many older adults to lose an important source of connection and engagement, leading to social isolation. To combat negative consequences, iN2L and a Florida AAA partnered on an innovative program providing iN2L tablets to AAA-supported older adults to use at home. The tablets have a simple interface, content specifically designed for older adults (e.g., games; music; movies), and video call capability. Participants included 51 independent older adults (mean age 77) and 39 family caregivers (mean age 59) of people with dementia. Participants completed phone surveys with AAA case managers at baseline and 3 months, including UCLA Loneliness Scale (3 item) and questions about their tablet experiences. Findings show positive trends for loneliness and well-being in both groups. At 3 months, lonely participants decreased from baseline by 25% for independent older adults and 18% for family caregivers. Over 80% of independent older adults agreed the tablet engages them in meaningful activities, provides daily enjoyment, and helps with relaxation. For family caregivers, 79% agreed the tablet is another tool in their caregiver toolkit and about 70% agreed the tablet adds daily enjoyment, helps with relaxation, and provides engagement in meaningful activities for their family member. Approximately 50% of caregivers felt happier, less stressed, and less irritable since using the tablets. This work has implications for the utility of technology in promoting engagement and connection, alleviating negative effects of social isolation, and the effectiveness of industry-AAA partnerships.

